# Diagnostic value of [^68^Ga]Ga-FAPI-04 in patients with colorectal cancer in comparison with [^18^F]F-FDG PET/CT

**DOI:** 10.3389/fonc.2022.1087792

**Published:** 2023-01-09

**Authors:** Xinfeng Lin, Yingjie Li, Shuailiang Wang, Yan Zhang, Xuetao Chen, Maomao Wei, Hua Zhu, Aiwen Wu, Zhi Yang, Xuejuan Wang

**Affiliations:** ^1^ Key Laboratory of Carcinogenesis and Translational Research (Ministry of Education/Beijing), NMPA Key Laboratory for Research and Evaluation of Radiopharmaceuticals (National Medical Products Administration), Department of Nuclear Medicine, Peking University Cancer Hospital and Institute, Beijing, China; ^2^ Department of Gastrointestinal Cancer Centre, Unit III, Key Laboratory of Carcinogenesis and Translational Research (Ministry of Education/Beijing), Peking University Cancer Hospital and Institute, Beijing, China

**Keywords:** fibroblast-activation protein inhibitor, colorectal cancer, fibroblast-activation protein, inhibitor, positron emission tomography

## Abstract

**Purpose:**

This study aimed to compare the diagnostic performance of [^68^Ga]Ga-FAPI-04 PET/CT and [^18^F]F-FDG PET/CT in primary and metastatic colorectal cancer (CRC) lesions.

**Methods:**

This single-center preliminary clinical study (NCT04750772) was conducted at the Peking University Cancer Hospital & Institute and included 61 participants with CRC who underwent sequential evaluation through PET/CT with [^18^F]F-FDG and [^68^Ga]Ga-FAPI-04. Their PET/CT images were analysed to quantify the uptake of the two tracers in the form of maximum standardised uptake (SUV_max_) values and target-to-background ratio (TBR), which were then compared using Wilcoxon’s signed-rank test. The final changes in the tumour–node–metastasis (TNM) stage of all participants were recorded.

**Results:**

Of all the participants, 21 were treatment naïve and 40 had been previously treated. In primary CRC lesions, the average TBRs of [^68^Ga]Ga-FAPI-04 and [^18^F]F-FDG were 13.3 ± 8.9 and 8.2 ± 6.5, respectively. The SUV_max_ of [^68^Ga]Ga-FAPI-04 in signet-ring/mucinous carcinomas (11.4 ± 4.9) was higher than that of [^18^F]F-FDG (7.9 ± 3.6) (*P* = 0.03). Both median SUV_max_ in peritoneal metastases and TBR in liver metastases of [^68^Ga]Ga-FAPI-04 were higher than those of [^18^F]F-FDG (5.2 vs. 3.8, *P* < 0.001; 3.7 vs. 1.9, *P* < 0.001, respectively). Compared with [^18^F]F-FDG PET/CT, clinical TNM staging based on [^68^Ga]Ga-FAPI-04 PET/CT led to upstaging and downstaging in 10 (16.4%) and 5 participants (8.2%), respectively. Therefore, the treatment options were changed in 13 participants (21.3%), including 9 with additional chemo/radiotherapy and/or surgery and others with avoidance or narrowed scope of surgery.

**Conclusion:**

[^68^Ga]Ga-FAPI-04 showed potential as a novel PET/CT tracer to detect lymph nodes and distant metastases, which improved CRC staging, thus prompting the optimisation or adjustment of treatment decisions.

## 1 Introduction

Colorectal cancer (CRC) was reported as the fifth most common cause of cancer-related deaths in the United States in 2022 (1). At diagnosis, 22% of patients with CRC have metastases, and 50% develop metastases during their lifetime. The overall 5-year survival of patients with CRC largely depends on the stage at presentation, varying from 80%–90% in the early stages to 13% in the advanced stage (2, 3). Currently, the key challenge is to establish optimal treatment plans according to the patients’ disease stage. Optimal imaging for CRC is crucial for accurate initial staging and the selection of primary therapy as well as during follow-up examinations for the accurate and timely detection of local recurrence and/or metastasis.

Non-invasive molecular imaging novel PET tracers is being increasingly used in the field of clinical oncology. Flourine-18 fluorodeoxyglucose ([^18^F]F-FDG) PET/CT, which uses altered glucose metabolism in cancer cells, is a valuable imaging modality in CRC management (4, 5). Compared with the routinely recommended conventional imaging modalities, [^18^F]F-FDG PET/CT can reflect cancer cells activity and the whole-body tumour burden. However, [^18^F]F-FDG PET/CT has several limitations, including low specificity, inability to detect small-volume lesions and lack of isotope uptake in mucinous and signet-ring cell carcinomas (6, 7). Tumour microenvironment imaging beyond the detection of tumour metabolism is a novel approach to elucidate *in vivo* tumour biology, with potential translational implications in clinical oncology. Fibroblast-activation protein (FAP) is a membrane-anchored peptidase that is highly expressed in cancer-associated fibroblasts (CAFs) in >90% of epithelial tumours, including CRC, and contributes to disease progression and worsening prognosis in various cancers (8–11). Several recently developed quinolone-based FAP inhibitors (FAPIs) coupled to chelators, including gallium-68 (^68^Ga)-labelled FAPI, are advantageous in staging and restaging many cancers, including peritonitis carcinomatosis, compared with [^18^F]F-FDG PET/CT (9–11). Koerber et al. (12) and Pang et al. (13) reported the avidity of [^68^Ga]Ga-FAPI in PET/CT imaging of CRC, supporting the potential use of FAP-targeted imaging in advanced CRC. We hypothesised that [^68^Ga]Ga-FAPI-04 PET/CT could contribute to CRC staging than conventional [^18^F]F-FDG PET/CT. Thus, we assessed the avidity of [^68^Ga]Ga-FAPI-04 in patients with CRC to compare the clinical impact of [^68^Ga]Ga-FAPI-04 PET/CT on tumour–node–metastasis (TNM) staging with that of standard-of-care [^18^F]F-FDG PET/CT imaging in participants with primary and recurrent/metastatic CRC.

## 2 Materials and methods

### 2.1 Participants

This preliminary clinical trial was approved by our Institutional Review Board (no. 2019KT95) and registered on ClinicalTrials.gov (NCT04750772). Written informed consent was obtained from all participants who were consecutively recruited from the study institution. The inclusion criteria were as follows: age >18 years, histologically confirmed CRC referral to the Nuclear Medicine Department for both [^18^F]F-FDG and [^68^Ga]Ga-FAPI-04 PET/CT scans for staging or restaging to aid optimal clinical decision making and provision of written informed consent. Pregnant or lactating women and those with severe liver or kidney dysfunction were excluded. The final cohort comprised 61 participants with CRC. The diagnosis was confirmed through surgery in 25 participants and through endoscopic biopsy in others. All suspicious metastatic lesions were confirmed by histopathology or follow-up for 3–6 months. Histopathology was served as a gold standard reference for the confirmation of the imaging findings by the means of rebiopsy or surgery. If pathological diagnosis was not applicable, conventional imaging (such as CT and MRI, etc.) follow-up for anatomical abnormalities of lesions was performed. Lesions were diagnosed of malignant based on any of the following follow-up criteria: 1) typical malignant features demonstrated by other imaging, especially the contrast-enhanced CT/MRI referred to as the first-line imaging. 2) posttreatment shrinkage or expansion of a suspicious lesion on follow-up imaging indicating improvement or progression, periodic plain CT/MRI scan used as the second-line imaging. 3) Density changes of lesions, such as cortical breakthrough for bone metastases. The finial observation of significant malignant features of suspicious lesions was the follow-up imaging end-point. The study flowchart of participant enrolment is presented in **Supplementary Figure 1**.

### 2.2 Synthesis of [^18^F]F-FDG and [^68^Ga]Ga-FAPI-04

[^18^F]F-FDG was manufactured in accordance with the standard method described by our laboratory using the coincidence [^18^F]F-FDG synthesis module. The FAPI precursor (DOTA-FAPI-04) was purchased from Huayi Technology Co., Ltd. (China), and synthesis and radiolabelling of [^68^Ga]Ga-FAPI-04 were performed as previously described (14). The radiochemical purity exceeded 95% for both [^18^F]F-FDG and [^68^Ga]Ga-FAPI-04. The final products underwent sterility testing before administration *via* intravenous injection.

### 2.3 PET/CT imaging

All enrolled participants underwent routine [^18^F]F-FDG PET/CT and subsequent [^68^Ga]Ga-FAPI-04 PET/CT within 1 week. All participants fasted for at least 6 h before [^18^F]F-FDG PET/CT, and a blood glucose level of <10 mmol/L was confirmed before tracer injection. Contrastingly, participants on a normal diet were intravenously injected with 1.85–2.96 MBq/kg [^68^Ga]Ga-FAPI-04 and underwent imaging using a hybrid PET/CT scanner (Biograph mCT Flow 64; Siemens Healthineers USA, Knoxville, TN, USA) after approximately 1 h. The acquisition was commenced in 6–8 bed positions (1 min/bed) covering the area between the top of the skull and upper thigh. Non-contrast-enhanced CT was performed using 100-mA modulation at 120 kV with a 3-mm slice thickness for attenuation correction and anatomical localisation. All data were transferred to the Syngo MultiModality Workplace (version VE40F; Siemens Healthineers) and reconstructed using the ordered subset expectation maximum algorithm to construct display images in the coronal, axial and sagittal planes.

### 2.4 Safety

Vital parameters, including blood pressure, heart rate, temperature and respiration rate, of all participants were carefully monitored during the examination. Any abnormal symptoms (e.g. allergy) were addressed as soon as possible.

### 2.5 Image analysis

All the images were reviewed by two groups of physicians with at least 10 years of experience in nuclear medicine and radiology. The physicians in group 1 (X.C. and M.W.) and group 2 (X.L. and X.W.) independently reviewed the [^18^F]F-FDG and [^68^Ga]Ga-FAPI-04 PET/CT images, respectively. Reference information from the other group and all other images and clinical data, including CT, MRI, endoscopic and pathological results, were absent. All differing opinions were interpreted and discussed within the groups until a consensus was reached. The inter-reader agreement within the two groups was expressed using the κ value.

Visual assessment was performed, and positive uptake was defined as focal tracer uptake exceeding background uptake. Circular volumes of interest within tumour lesions and healthy tissues were used to quantify radiotracer biodistribution. Tracer uptake was quantified using maximum standardised uptake (SUV_max_) values, which was measured by drawing regions of interest around the tumours on transaxial slices that were automatically adapted to a three-dimensional volume of interest with the system software at an 80% isocontour. The normal organs were evaluated using a 1–2-cm-diameter circular sphere. Primary lesions, lymph nodes and distant metastases were analysed. The lymph nodes were classified according to their location as cervical–supraclavicular, thoracic, abdominal and pelvic. The target-to-background ratios (TBRs) of the primary lesions; lymph nodes; and liver, lung, bone and peritoneal metastatic tumours were calculated (the normal transverse colon without physiological uptake, blood pool of the aorta, normal liver tissue, normal lung tissue, L5 and normal mesenterium were used as backgrounds, respectively).

We used TNM classification based on the National Comprehensive Cancer Network *(NCCN)* guidelines (15, 16). In all participants, changes in the TNM stage, metastases localisation and previous oncologic or radio-oncologic management history were recorded.

### 2.6 Statistical analyses

All statistical analyses were performed using SPSS 23.0 (IBM, Armonk, NY, USA). Inter-reader agreement was evaluated using Kappa test. The uptakes of positive lesions in [^18^F]F-FDG and [^68^Ga]Ga-FAPI-04 PET/CT were compared using Wilcoxon’s signed-rank test. SUV_max_ and TBR were the main parameters for evaluating the two PET/CT scans, and normally distributed and skewed variables were expressed as means (95% confidence intervals) and medians (ranges), respectively. A two-tailed *P* value of <0.05 was considered statistically significant.

## 3 Results

### 3.1 Participant characteristics

**Table 1** summarises the clinical characteristics of the 61 participants. The median age of the participants was 62 (range, 32–81) years, and 42 (68.9%) participants were men. The most common histologic grade was moderate differentiation in 35 (57.4%) participants, whereas 8 (13.1%) and 7 (11.5%) participants had mucinous/signet-ring cell carcinoma and adenocarcinoma with a mucinous component, respectively. Overall, 21 (34.4%) participants underwent PET/CT for initial assessment and staging; the remaining 40 (65.6%) underwent PET/CT for restaging or therapeutic evaluation.

**Table 1:** Participants’ characteristics.

Characteristic	Value
Participants (*n*)	61
Age (years), median (range)	62 (32–81)
Sex (male:female)	42:19
Colon cancer	26 (42.6%)
Rectal cancer	35 (57.4%)
Treatment status	
Treatment-naïve	21
Neoadjuvant treatment	15
Chemotherapy	5
Radiotherapy	3
Chemotherapy + radiotherapy	7
Chemotherapy/radiotherapy after surgery	25
Pathology	
Adenocarcinoma (poorly differentiated)	8
Adenocarcinoma (moderately differentiated)	35
Adenocarcinoma (well-differentiated)	3
Mucinous/signet-ring cell carcinoma	8
Adenocarcinoma with mucinous component	7
Purpose of PET/CT	
Staging	21
Restaging/therapeutic evaluation	40

CT, computed tomography; PET, positron emission tomography.

### 3.2 Safety

All participants tolerated [^68^Ga]Ga-FAPI-04 PET/CT without any complications. No signs of drug-related side effects were reported during the entire observation period of >5 h.

### 3.3 Distribution

**Figure 1:**
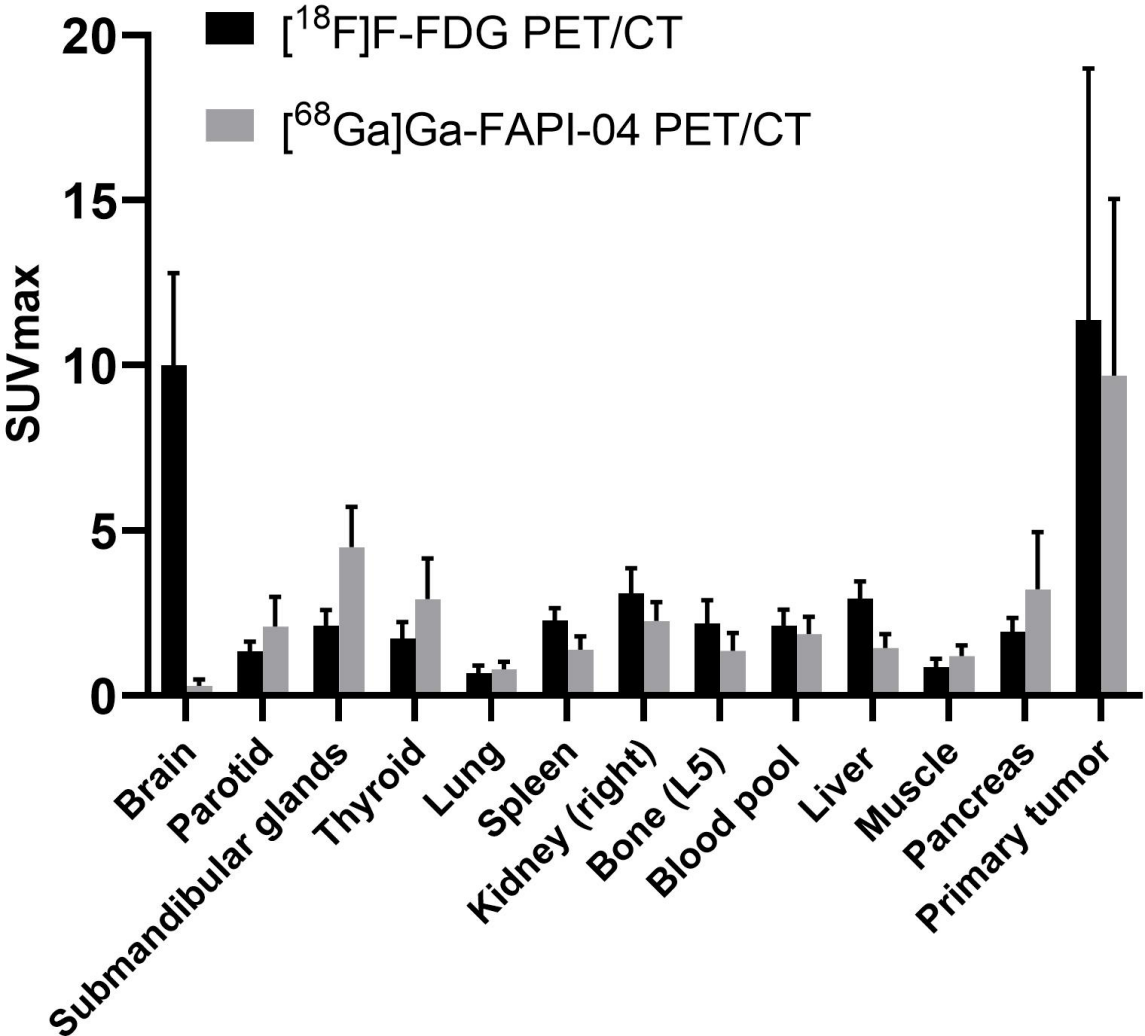
PET-based biodistribution analysis of 61 participants evaluated using [^68^Ga]Ga-FAPI-04 PET/CT and [^18^F]F-FDG PET/CT 1 h after tracer injection.

The inter-reader agreement between groups 1 and 2 was nearly perfect, and the *κ* value was >0.8 (**Supplementary Tables 1–4**). SUV_max_ was determined for normal tissues/organs and primary tumours after [^68^Ga]Ga-FAPI-04 and [^18^F]F-FDG PET/CT, which were sequentially performed for all the participants. [^68^Ga]Ga-FAPI-04 activity was significantly lower than [^18^F]F-FDG activity in several normal organs (*P* < 0.001), especially the brain (SUV_max_, 0.3 ± 0.2 vs. 10.0 ± 2.8, *P* < 0.001) and liver (SUV_max_, 1.4 ± 0.4 vs. 2.9 ± 0.5, *P* < 0.001), leading to significantly high TBRs of >2 in both organs (*P* < 0.001). Further, [^68^Ga]Ga-FAPI-04 uptake was higher than [^18^F]F-FDG uptake in the salivary and thyroid glands and the pancreas (*P* < 0.001 for all). The detailed distribution of [^18^F]F-FDG and [^68^Ga]Ga-FAPI-04 uptakes is presented in **Figure 1**.

### 3.4 Primary tumours

The histopathological data of the primary tumours were available for all treatment-naïve participants (*n* = 21) and for those who received neoadjuvant treatment (*n* = 15). Only 1 of these 36 participants had two primary lesions. Therefore, 37 primary lesions were measured. The sensitivity was 100% (37/37) for both [^68^Ga]Ga-FAPI-04 and [^18^F]F-FDG PET/CT. The average SUV_max_ and median SUV_max_ (range) of all primary lesions were 9.7 ± 5.4 and 9.7 (2.0–25.5), respectively, on [^68^Ga]Ga-FAPI-04 PET/CT and 11.4 ± 7.6 and 10.3 (2.4–35.1), respectively, on [^18^F]F-FDG PET/CT (*P* = 0.09). The average TBR of all 37 primary lesions was significantly higher on [^68^Ga]Ga-FAPI-04 PET/CT than on [^18^F]F-FDG PET/CT (13.3 ± 8.9 and 8.2 ± 6.5, respectively; *P* < 0.001). The average SUV_max_ values for [^68^Ga]Ga-FAPI-04 and [^18^F]F-FDG were 12.3 ± 4.6 and 14.1 ± 7.3, respectively, in the treatment-naïve group (*P* = 0.21) and 5.9 ± 4.1 and 7.4 ± 6.5, respectively, in the neoadjuvant radio-chemotherapy group (*P =* 0.18). The average TBR of the treatment-naïve lesions was significantly higher on [^68^Ga]Ga-FAPI-04 PET/CT than on [^18^F]F-FDG PET/CT (17.6 ± 8.5 vs. 10.5 ± 7.2, *P* = 0.002), whereas the average TBR was not different between the imaging modalities for the post-treatment lesions (7.0 ± 5.0 vs. 5.0 ± 3.4, *P* = 0.061).

**Figure 2:**
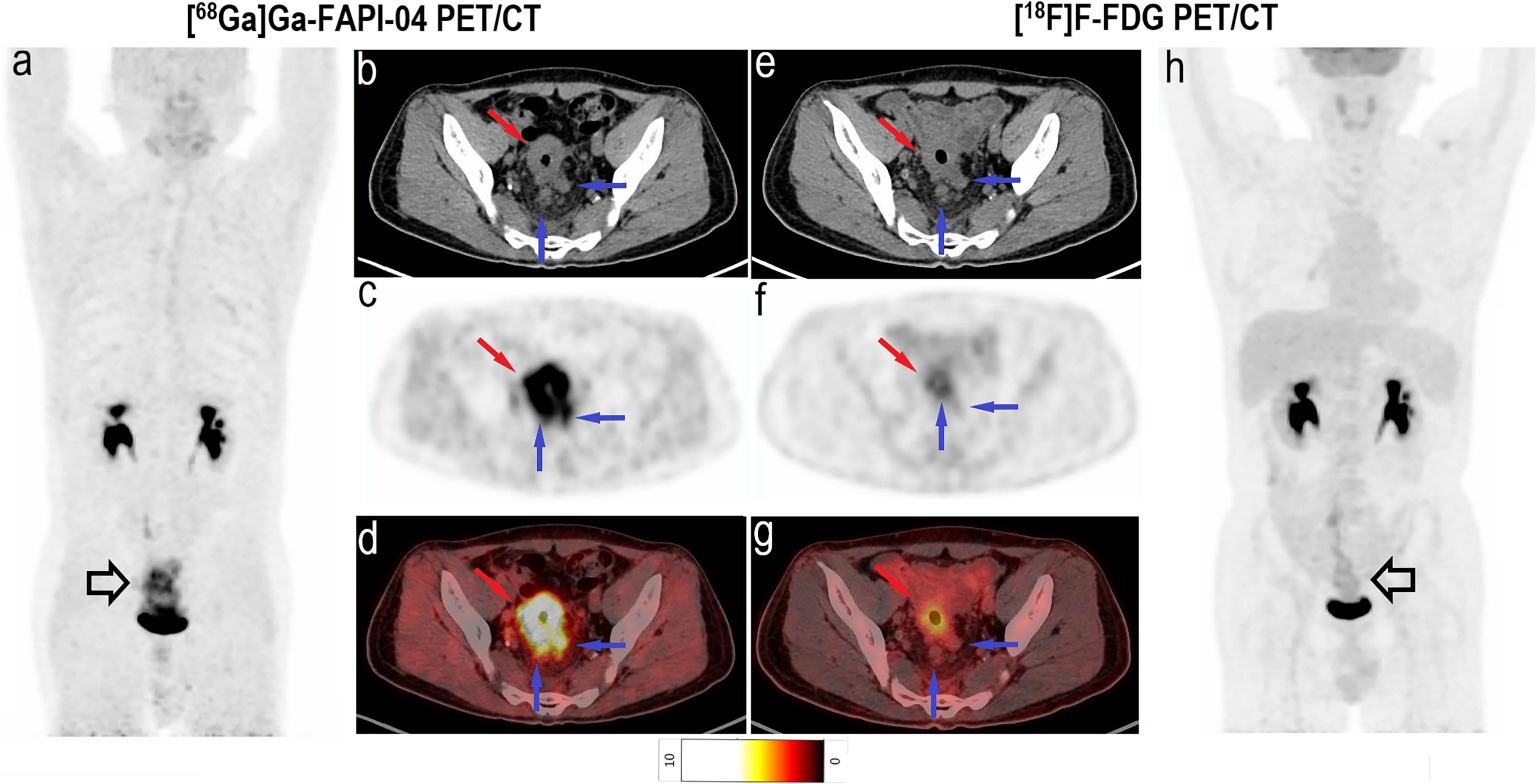
Images of a 49-year-old man with rectal mucinous carcinoma for staging. **(A)** Mean intensity projection images of [^68^Ga]Ga-FAPI-04 PET/CT scan. **(B–D)** Axial CT, PET and fused images of [^68^Ga]Ga-FAPI-04 PET/CT scan. **(E–G)** Axial CT, PET and fused images of [^18^F]F-FDG PET/CT scan. **(H)** Mean intensity projection images of [^18^F]F-FDG PET/CT scan. Compared with [^18^F]F-FDG PET/CT, [^68^Ga]Ga-FAPI-04 PET/CT exhibits the primary lesion (hollow black and red arrows) and suspicious lymph nodes (hollow black and blue arrows) more clearly because of the higher tracer uptake. The SUV_max_ of primary lesions was 14.6 for [^68^Ga]Ga-FAPI-04 and 4.4 for [^18^F]F-FDG PET/CT. The [^68^Ga]Ga-FAPI-04 PET/CT images also depict more intense tracer uptake in suspicious lymph nodes than [^18^F]F-FDG PET/CT images, with SUV_max_ values of 5.6–6.0 and 1.5–2.0, respectively.

The analysis of treatment-naïve primary tumours revealed that the avidity of [^68^Ga]Ga-FAPI-04 (11.4 ± 4.9) was significantly higher than that of [^18^F]F-FDG (7.9 ± 3.6) in signet-ring/mucinous carcinomas (*P* = 0.03; **Figure 2**). Additionally, [^68^Ga]Ga-FAPI-04 uptake was significantly lower than [^18^F]F-FDG uptake in poorly differentiated carcinomas (average SUV_max_, 12.7 ± 3.7 vs. 18.1 ± 4.1; *P* = 0.04). There was also a significant difference in the SUV_max_ of primary lesions between [^68^Ga]Ga-FAPI-04 and [^18^F]F-FDG PET/CT among well-differentiated and moderately differentiated carcinomas (average SUV_max_, 10.8 ± 3.0 vs. 16.2 ± 8.6; *P* = 0.025). Although the uptake of [^68^Ga]Ga-FAPI-04 was higher in poorly differentiated carcinomas (12.7 ± 3.7) than in moderately-well differentiated carcinomas (10.8 ± 3.0), no significant difference was noted between them (*P* = 0.074). Interestingly, in the neoadjuvant chemotherapy group, there was no significant difference in the SUV_max_ of primary lesions between [^68^Ga]Ga-FAPI-04 and [^18^F]F-FDG PET/CT among all carcinoma types (*P* = 0.182).

### 3.5 Changes in TNM stage

Compared with staging based on [^18^F]F-FDG PET/CT, [^68^Ga]Ga-FAPI-04 PET/CT revealed additional findings in 34 of the 61 participants, which led to changes in staging in 15 participants. Particularly, 6 (28.6%) of the 21 treatment-naïve participants were upstaged and 2 (9.5%) were downstaged (**Table 2**).

**Table 2:** Comparison of FDG PET-based and FAPI PET-based TNM staging of 34 CRC participants with additional findings.

Number	Treatment status	TNM stage (FDG PET-based)	TNM stage (FAPI PET-based)	Additional finding in FAPI PET	Reference standard	Change in staging
2	Treatment-naïve	T4aN2bM0 (III B)	T4aN2bM1c (IV C)	Peritoneal metastasis	Contrast-enhanced MRI	Upstage
4	Treatment-naïve	T4aN2bM1c (IV C)	T4aN2bM1c (IV C)	2 LNs	Shrinkage after treatment (contrast CT)	None
5	Treatment-naïve	T1/2N0M1a (IV A)	T1/2N0M0 (I)	No rectal metastasis	Endoscopic biopsy	Downstage
6	Treatment-naïve	T4aN2aM1c (IV C)	T4aN2aM1c (IV C)	2 LNs	Shrinkage after treatment (contrast CT)	None
11	Neoadjuvant treatment	yT0N0M1a (IV A)	yT0N0M0	No small intestinal metastasis	Endoscopic biopsy	Downstage
15	Treatment-naïve	T3N1bM1a (IV A)	T3N1bM1a (IV A)	1 LN	Shrinkage after treatment (contrast CT)	None
16	Treatment-naïve	T4bN2bM0 (III C)	T4bN2bM0 (III C)	5 LNs	Shrinkage after treatment (contrast CT)	None
17	Treatment-naïve	T4aN1bM0 (III B)	T4aN1bM0 (III B)	1 LN	Shrinkage after treatment (contrast CT)	None
20	Treatment-naïve	T3N2aM1a (IV A)	T3N2aM1a (IV A)	1 LN	Shrinkage after treatment (contrast CT)	None
21	Treatment-naïve	T4aN1bM0 (III B)	T4aN1bM0 (III B)	1 LN	Surgery	None
22	Treatment-naïve	T3N1bM0 (III B)	T3N1bM1c (IV C)	1 LN + Peritoneal metastasis	Surgery	Upstage
23	Treatment-naïve	T3N1bM0 (III B)	T3N1bM0 (III B)	1 LN	Surgery	None
24	Post-operation	M1	M0	No metastatic recurrence in anastomotic stoma	Endoscopic biopsy	Downstage
26	Treatment-naïve	T3N1bM0 (III B)	T3N1bM0 (III B)	1 LN	Shrinkage after treatment (contrast CT)	None
27	Treatment-naïve	T3N0M0 (II A)	T3N1aM0 (III B)	1 LN	Surgery	Upstage
28	Treatment-naïve	T3N1cM0 (III B)	T3N1cM1a (IV A)	1 LN + Left acetabulum metastasis	Staging surgery and shrinkage after treatment (contrast CT)	Upstage
30	Neoadjuvant treatment	yT3N1aM0 (III B)	yT3N1bM0 (III B)	1 LN	Shrinkage after treatment (contrast CT)	None
33	Treatment-naïve	T4bN2bM0 (III C)	T4bN2bM0 (III C)	2 LNs	Shrinkage after treatment (contrast CT)	None
35	Treatment-naïve	T4aN1aM0 (III B)	T4aN2bM0 (III C)	7 LNs	Shrinkage after treatment (contrast CT)	Upstage
36	Treatment-naïve	T4aN2aM0 (III C)	T4aN2aM1a (IV A)	2 LNs + Liver metastasis	Contrast-enhanced MRI	Upstage
37	Post-operation	M1c	M1c	No 2 right axillary and internal mammary LN metastases	No change in size (follow-up by CT)	None
38	Treatment-naïve	T4aN1aM0 (III B)	T4aN1bM0 (III B)	1 LN	Surgery	None
40	Post-operation	M1c	M1c	No 1 right hilar LN metastasis	No change in size (follow-up by CT)	None
41	Post-operation	M1b	M0	No 7 mediastinal and hilar LN metastases	No change in size (follow-up by CT)	Downstage
44	Post-operation	M1c	M1c	1 left hilar LN metastasis	Shrinkage after treatment (contrast CT)	None
46	Post-operation	M1c	M1c	No metastatic recurrence in anastomotic stoma	Endoscopic biopsy	None
47	Post-operation	M1b	M1c	Peritoneal metastases	Expansion after drug resistance	Upstage
49	Post-operation	M0	M1a	Left 11th rib metastasis	Changes in bone density (contrast CT)	Upstage
50	Post-operation	M1a	M1c	Peritoneal metastases (liver capsule)	Contrast-enhanced MRI	Upstage
52	Post-operation	M1c	M1c	> 7 peritoneal metastases	Shrinkage after treatment (contrast CT)	None
53	Neoadjuvant treatment	yT3N0M0 (II A)	yT3N1bM1b (IV B)	9 LNs	Shrinkage after treatment (contrast CT)	Upstage
55	Treatment-naïve	T3N0M1a (IV A)	T3N0M0 (II A)	No 1 left hilar LN metastasis	No change in size (follow-up by CT)	Downstage
58	Neoadjuvant treatment	yT1/2N0M1b (IV B)	yT1/2N0M1a (IV A)	No 4 cervical LN metastases	No change in size (follow-up by CT)	None
59	Post-operation	M1c	M1c	No metastatic recurrence in anastomotic stoma	Endoscopic biopsy	None

T1/2, inability to differentiate T1 and T2 stages using PET/CT; LN, lymph node; FDG, fluorodeoxyglucose; FAPI, fibroblast-activation protein inhibitor; SUV_max_, maximum standardised uptake value; TNM, tumour–node–metastasis.

**Figure 3:**
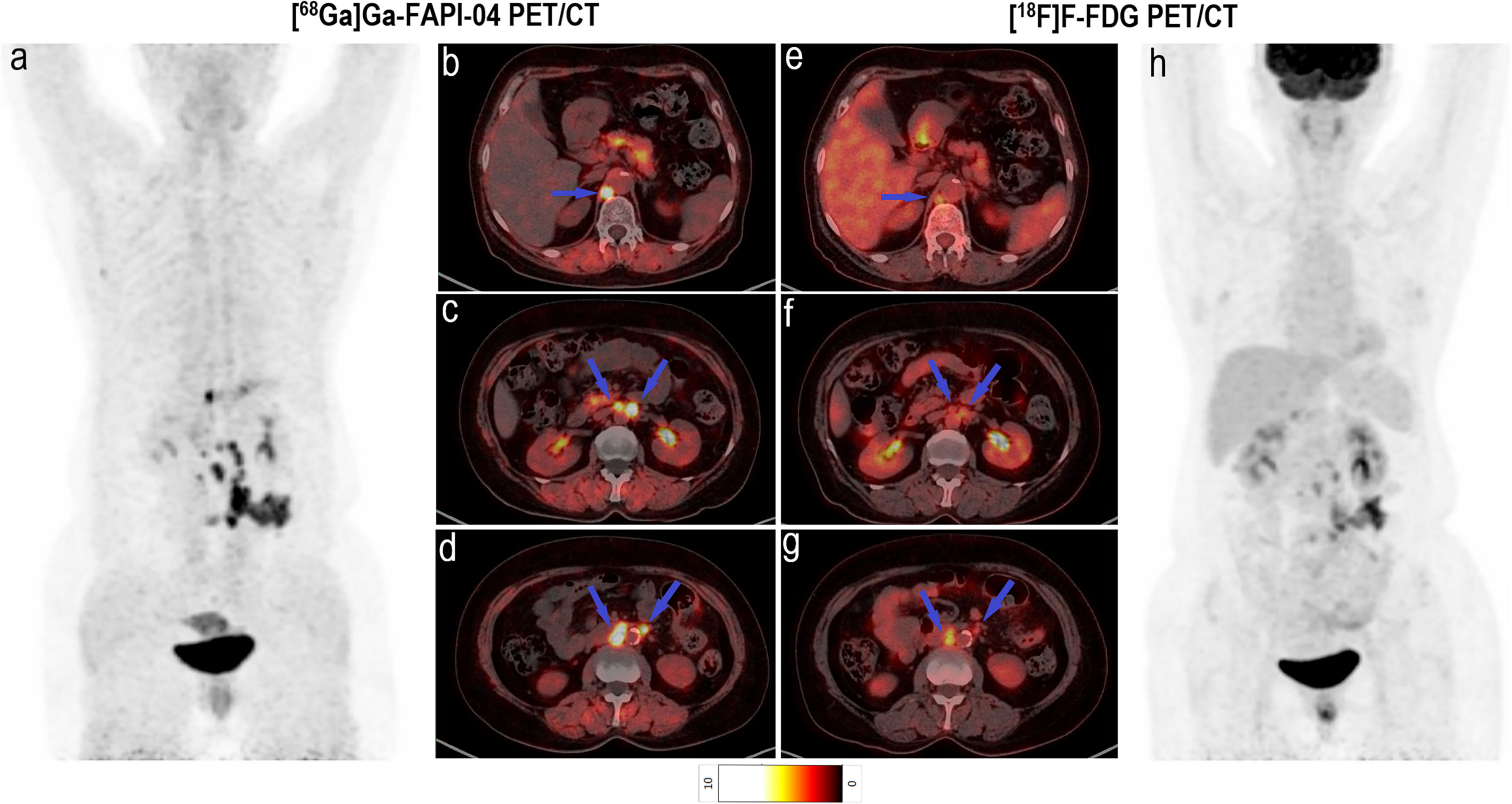
Images of a 64-year-old woman with colon cancer for staging. **(A)** Mean intensity projection images of [^68^Ga]Ga-FAPI-04 PET/CT scans. **(B–D)** Axial fused images of suspicious lymph nodes at different levels of the body in [^68^Ga]Ga-FAPI-04 PET/CT. **(E–G)** Axial fused images of suspicious lymph nodes at different levels of the body in [^18^F]F-FDG PET/CT. **(H)** Mean intensity projection images of [^18^F]F-FDG PET/CT scan. [^68^Ga]Ga-FAPI-04 uptake (blue arrows in **B–D**) was higher than [^18^F]F-FDG uptake (blue arrows in **E–G**) in both the diaphragmatic and retroperitoneal metastatic lymph nodes, with SUV_max_ values of 5.9–12.0 and 3.4–4.3, respectively.

Among all participants who were upstaged based on [^68^Ga]Ga-FAPI-04 PET/CT findings (10/61, 16.4%), the changes were based on the detection of new or additional distant metastases in one or more organ systems. All additional findings were confirmed through biopsy or surgery (10/34, 29.4%) or through other conventional imaging modalities (24/34, 70.6%). Moreover, in 16 participants, new lymph node metastases were detected but did not lead to changes in the stage (**Table 2**). The median uptake of [^68^Ga]Ga-FAPI-04 was higher than that of [^18^F]F-FDG in both abdominal (6.4 vs. 4.2, *P* < 0.001) and pelvic lymph nodes (4.6 vs. 2.7, *P* < 0.001; **Table 3**, **Figure 3**). The TBRs of both abdominal (3.5 vs. 2.1, *P* < 0.001) and pelvic lymph nodes (2.9 vs. 1.2, *P* < 0.001) were also significantly higher in [^68^Ga]Ga-FAPI-04 PET/CT images than in [^18^F]F-FDG PET/CT images. However, the median uptake (3.0 vs. 4.7, *P* < 0.001) and TBR (1.4 vs. 2.4, *P* < 0.001) of [^68^Ga]Ga-FAPI-04 were lower than those of [^18^F]F-FDG in thoracic lymph nodes (**Table 3**).

**Table 3:** Comparison of [^18^F]F-FDG and [^68^Ga]Ga-FAPI-04 uptake in colorectal tumour sites.

Tumour sites and parameters	[^18^F]F-FDG PET/CT	[^68^Ga]Ga-FAPI-04 PET/CT	*P-*value
Primary tumour			
No. of lesions (participants)	37 (36)	37 (36)	
Mean SUV_max_ (95% CI)	11.4 (9.1, 13.8)	9.7 (8.0, 11.4)	0.09
Mean TBR (95% CI)	8.2 (6.4, 10.6)	13.3 (10.5, 16.2)	<0.001
Involved lymph nodes			
**Cervical–supraclavicular**			
No. of lesions (participants)	6 (2)	4 (2)	
Median SUV_max_ (range)	3.2 (2.1–4.3)	2 (1.8–2.5)	0.027
Median TBR (range)	1.6 (1.3–2.3)	1.2 (1.1–1.5)	0.027
**Thoracic** ^*^			
No. of lesions (participants)	26 (13)	18 (13)	
Median SUV_max_ (range)	4.7 (2.4–10.2)	3.0 (1.9–12.9)	<0.001
Median TBR (range)	2.4 (1.0–4.9)	1.4 (1.0–5.4)	<0.001
**Abdominal** ^†^			
No. of lesions (participants)	26 (9)	38 (9)	
Median SUV_max_ (range)	4.2 (2.1–9.4)	6.4 (2.7–20.5)	<0.001
Median TBR (range)	2.1 (1.2–6.3)	3.5 (1.5–13.7)	<0.001
**Pelvic** ^§^			
No. of lesions (participants)	37 (15)	72 (15)	
Median SUV_max_ (range)	2.7 (2.0–6.6)	4.6 (2.5–17.6)	<0.001
Median TBR (range)	1.2 (0.7–3.3)	2.9 (1.5–9.8)	<0.001
Involved distant lesions			
**Liver**			
No. of lesions (participants)	16 (9)	30 (9)	
Median SUV_max_ (range)	4.6 (2.5–9.9)	3.9 (1.7–12.2)	0.951
Median TBR (range)	1.9 (0.6–4.7)	3.7 (1.6–7.6)	<0.001
**Lung**			
No. of lesions (participants)	24 (9)	23 (9)	
Median SUV_max_ (range)	2.1 (0.9–11.0)	2.1 (1.0–11.2)	0.484
Median TBR (range)	4.1 (1.3–22.0)	3.5 (1.2–22.4)	0.017
**Bone**			
No. of lesions (participants)	6 (5)	8 (5)	
Median SUV_max_ (range)	5.5 (4.0–8.8)	7.9 (2.8–14.0)	0.036
Median TBR (range)	3.4 (2.9–5.0)	8.7 (3.1–10.9)	0.017
**Peritoneum**			
No. of lesions (participants)	45 (20)	60 (20)	
Median SUV_max_ (range)	3.8 (1.1–16.4)	5.2 (2.1–12.6)	<0.001
Median TBR (range)	3.8 (1.8–29.0)	6.9 (2.6–31.5)	<0.001
**Brain**			
No. of lesions (participants)	1 (1)	1 (1)	
Median SUV_max_ (range)	6.9 (6.9)	2.1 (2.1)	NA
Median TBR (range)	0.9 (0.9)	21 (21)	NA

*Lymph nodes in the thoracic regions include mediastinal or/and hilar, axillary and internal mammary lymph nodes.

†Lymph nodes in the abdominal regions include para-aortic, retroperitoneal and celiac lymph nodes.

§Lymph nodes in the pelvic regions include pelvic, iliac and inguinal lymph nodes; ^18^F, fluorine 18; ^68^Ga, gallium 68; FDG, fluorodeoxyglucose; FAPI, fibroblast-activation protein inhibitor; SUV_max_, maximum standardised uptake value; NA, not applicable.

**Figure 4:**
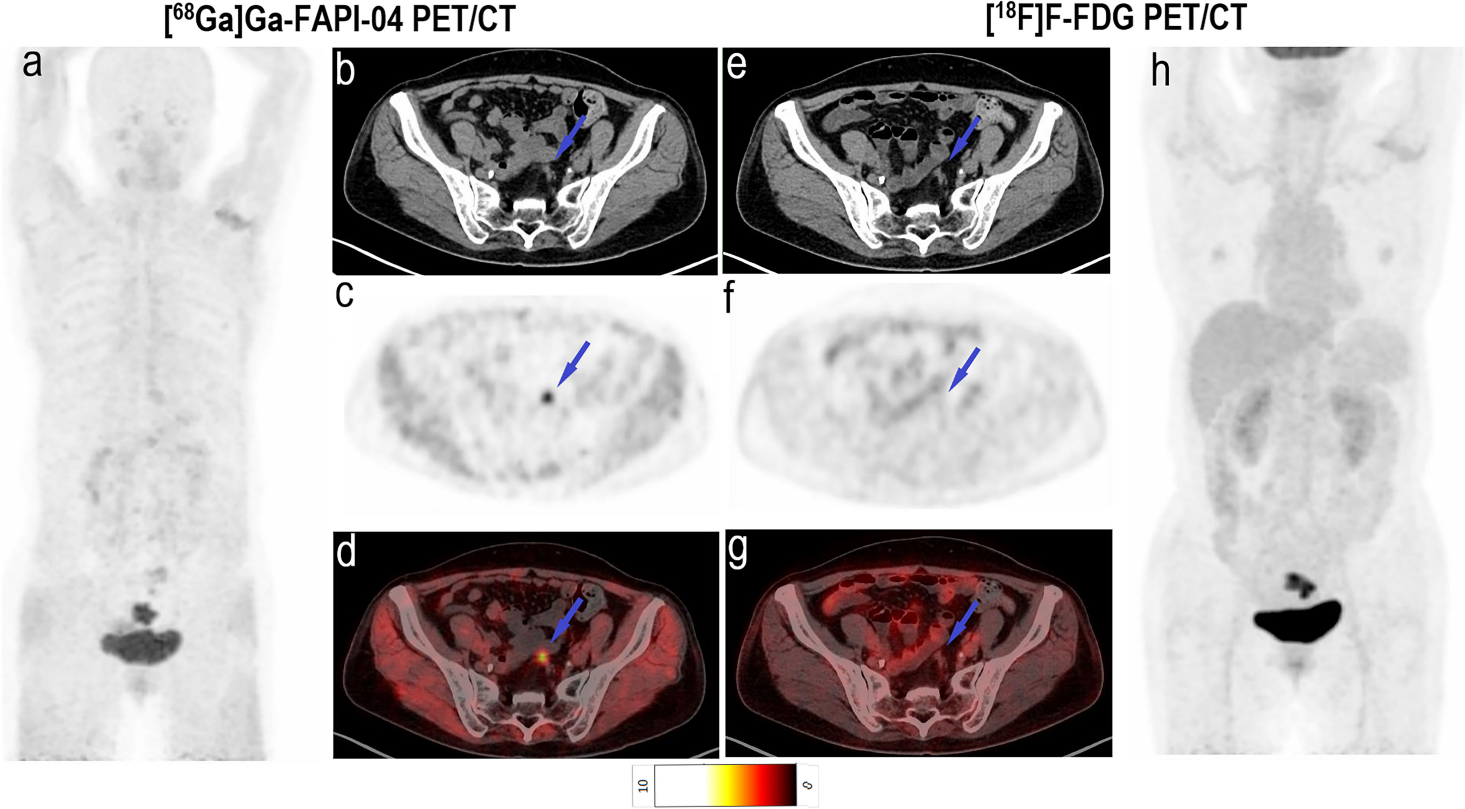
Images of a 64-year-old woman with rectal cancer for staging. **(A)** Mean intensity projection images of [^68^Ga]Ga-FAPI-04 PET/CT scan. **(B–D)** Axial CT, PET and fused images of [^68^Ga]Ga-FAPI-04 PET/CT scan. **(E–G)** Axial CT, PET and fused images of [^18^F]F-FDG PET/CT scan. **(H)** Mean intensity projection images of [^18^F]F-FDG PET/CT scan. Pelvic peritoneal carcinoma is distinctly observed in [^68^Ga]Ga-FAPI-04 PET/CT images (blue arrows in **b–d**) because of intensive tracer uptake (SUV_max_ 5.5). Conversely, little [^18^F]F-FDG (SUV_max_ 1.1) uptake results in the small lesion being hardly detectable (blue arrows in **E–G**).

**Figure 5:**
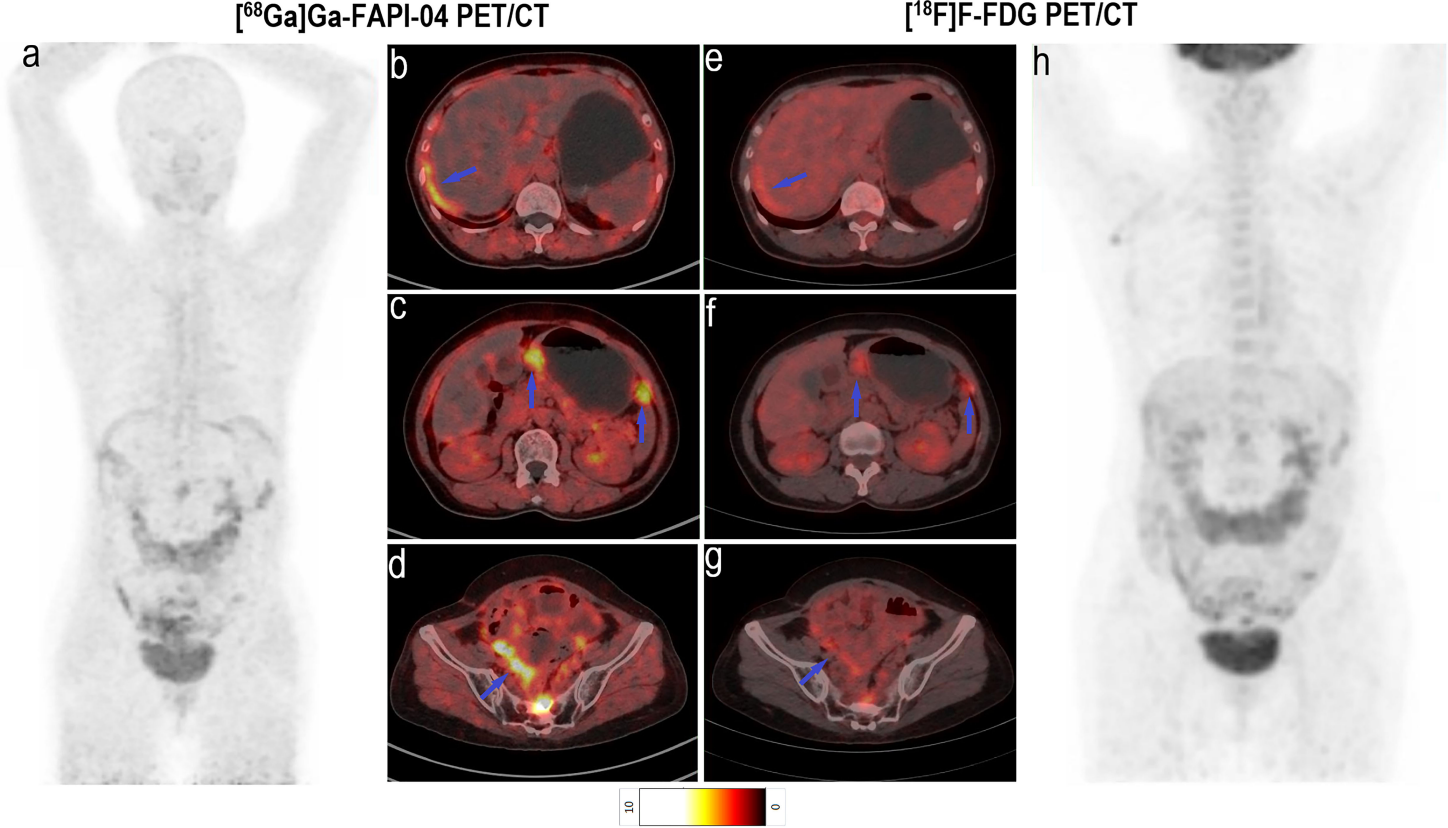
Images of a 60-year-old woman with colon cancer and metastatic peritoneal carcinoma for restaging after treatment. **(A)** Mean intensity projection images of [^68^Ga]Ga-FAPI-04 PET/CT scans. **(B–D)** Axial fused images of metastatic peritoneal carcinomas at different body levels in [^68^Ga]Ga-FAPI-04 PET/CT. **(E–G)** Axial fused images of metastatic peritoneal carcinomas at different body levels in [^18^F]F-FDG PET/CT. **(H-h)** Mean intensity projection images of [^18^F]F-FDG PET/CT scan. [^68^Ga]Ga-FAPI-04 uptake (blue arrows in **B–D**) was higher than the [^18^F]F-FDG uptake (blue arrows in **E–G**) in peritoneal carcinomas, and SUV_max_ values for [^68^Ga]Ga-FAPI-04 and [^18^F]F-FDG PET/CT were 4.6–8.8 and 3.1–3.5, respectively.

Imaging with [^68^Ga]Ga-FAPI-04 PET/CT led to upstaging based on the detection of peritoneal and bone metastases in four and two participants, respectively (**Table 2**). In all 20 participants, the SUV_max_ (5.2 vs. 3.8, *P* < 0.001) and TBR (6.9 vs. 3.8, *P* < 0.001) of [^68^Ga]Ga-FAPI-04 were higher than those of [^18^F]F-FDG in peritoneal metastases (**Table 3**). Compared with the [^18^F]F-FDG PET/CT images, the peritoneal metastases were clearly visible in the [^68^Ga]Ga-FAPI-04 PET/CT images (**Figures 4**, **5**). There was no significant difference in SUV_max_ between [^68^Ga]Ga-FAPI-04 and [^18^F]F-FDG in positive lung lesions (*P* = 0.484), but the TBR of [^68^Ga]Ga-FAPI-04 was significantly lower than that of [^18^F]F-FDG (*P* = 0.017; **Table 3**).

**Figure 6:**
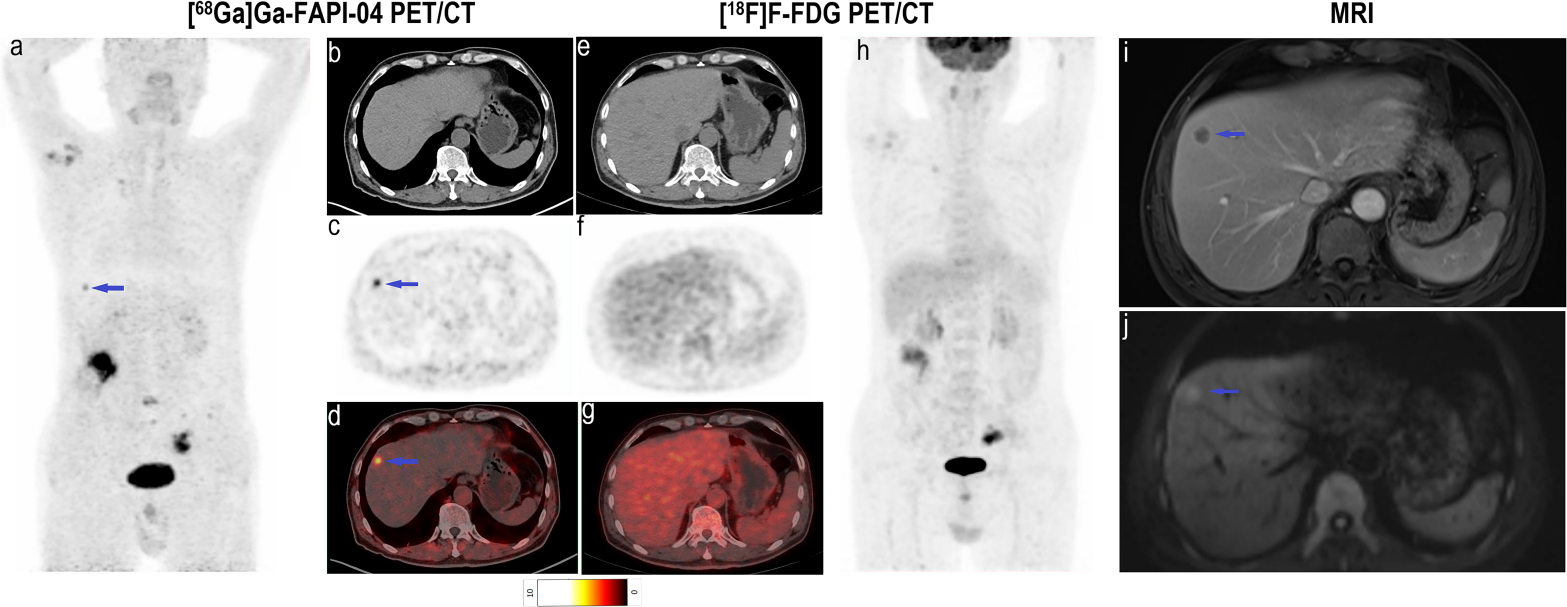
Images of a 63-year-old man with colon cancer for staging. **(A)** Mean intensity projection images of [^68^Ga]Ga-FAPI-04 PET/CT scans. **(B–D)** Axial CT, PET and fused images of [^68^Ga]Ga-FAPI-04 PET/CT scan. **(E–G)** Axial CT, PET and fused images of [^18^F]F-FDG PET/CT scan. **(H)** Mean intensity projection images of [^18^F]F-FDG PET/CT scan. **(I, F)** Images of contrast-enhanced MRI, T2WI (delay phase) and DWI (b = 1000). The suspicious metastatic lesion in the right liver lobe is clearly visible in [^68^Ga]Ga-FAPI-04 PET/CT images (blue arrows in **A–D**; SUV_max_ 5.0) but absent in [^18^F]F-FDG PET/CT images. Its presence was confirmed by contrast-enhanced MRI (blue arrows in **I**, **J**).

Although there was no significant difference in the SUV_max_ of [^68^Ga]Ga-FAPI-04 and [^18^F]F-FDG in positive liver lesions (3.9 vs. 4.6, *P* = 0.951), the number of positive liver lesions detected using [^68^Ga]Ga-FAPI-04 PET/CT was higher than that detected by [^18^F]F-FDG PET/CT because of the lower background SUV_max_ of [^68^Ga]Ga-FAPI-04. Further, 30 positive liver lesions detected using [^68^Ga]Ga-FAPI-04 PET/CT were confirmed as metastases through surgery/biopsy and other imaging modalities. Only 16 of these positive liver lesions were detected by [^18^F]F-FDG PET/CT (53.3%, 16/30). The TBR of [^68^Ga]Ga-FAPI-04 was significantly higher than that of [^18^F]F-FDG (3.7 vs. 1.9, *P* < 0.001), and the liver metastases were clearly visible in [^68^Ga]Ga-FAPI-04 PET/CT images and finally demonstrated using contrast-enhanced MRI (**Figure 6**). **Table 3** presents the detailed comparison of liver metastases detected using [^68^Ga]Ga-FAPI-04 and [^18^F]F-FDG PET/CT.

The final staging changes in 15 participants were verified based on the reference standards; thus, 13 participants’ treatment options were changed from their pre-examination or originally planned regimens based on the changed stage. Chemo/radiotherapy and/or surgery was added in nine participants (#2, #22, #28, #35, #47, #49, #50, #53 and #55), and surgery was avoided or its scope was narrowed in the other participants (#5, #11, #24 and #41).

## 4 Discussion

FAP is an excellent target for tumour stroma, and ^68^Ga-FAPIs, as newer imaging tracers, present a promising alternative to [^18^F]F-FDG. This preliminary clinical study investigated the avidity of [^68^Ga]Ga-FAPI-04 in CRC and explored the potential utility of [^68^Ga]Ga-FAPI-04 PET/CT as the sole imaging modality for assessing primary and recurrent/metastatic CRC. Our analyses indicated that [^68^Ga]Ga-FAPI-04 PET/CT improved tumour staging in patients with CRC owing to favourable tumour/background activity and low tracer uptake in the gastrointestinal tract. Moreover, signet-ring/mucinous carcinomas accumulated more [^68^Ga]Ga-FAPI-04 than [^18^F]F-FDG, whereas FAPI avidity was lower than FDG avidity in poorly differentiated carcinomas.

As key constituents of the tumour stroma, CAFs can support the immunosuppressive microenvironment, tumour cell growth, progression and metastatic potential. Expressed by CAFs, FAP is an attractive diagnostic and therapeutic target (8, 10, 17). [^68^Ga]Ga-FAPI-04 PET/CT is characterised by high tumour/background activity and is more sensitive than [^18^F]F-FDG PET/CT for identifying primary gastrointestinal carcinoma lesions (12, 13).

The origin, number and distribution of FAP-expressing CAFs and the number of FAP molecules per cell may differ among tumours. Mona et al. (18) reported a strong correlation between tumour [^68^Ga]Ga-FAPI-46 uptake intensity and histopathological FAP expression in colon cancer. We expected variations in intra-tumoural tracer distribution in treatment-naïve patients with specific histopathologic types of CRC. In this study, we demonstrated additional FAP expression in signet-ring/mucinous carcinomas, which normally exhibit low [^18^F]F-FDG uptake (6, 7, 19). The results revealed that [^68^Ga]Ga-FAPI-04 PET/CT would have a lower false-negative rate than [^18^F]F-FDG PET/CT in detecting primary and metastatic CRC lesions. Although Solano-Iturri et al. (20) reported that CRC tissues with poor differentiation exhibited a higher percentage of FAP staining than those with moderately-well differentiation, the poorly differentiated carcinomas exhibited moderately higher [^68^Ga]Ga-FAPI-04 uptake without significant differences between these subtypes in our study. Moreover, we found that poorly differentiated carcinomas exhibited significantly lower [^68^Ga]Ga-FAPI-04 uptake than [^18^F]F-FDG uptake, although this subtype showed avidity for both [^68^Ga]Ga-FAPI-04 and [^18^F]F-FDG.

The TNM classification provides standard guidelines to classify the extent of cancer metastasis. The degree of tumour progression and invasion at the time of surgical resection as well as patient outcomes are estimated on the basis of this staging system for CRC. This study demonstrated that [^68^Ga]Ga-FAPI-04 PET/CT could detect both primary tumours and metastases arising from CRC. The sensitivity was 100% for both [^68^Ga]Ga-FAPI-04 and [^18^F]F-FDG PET/CT, and no significant differences in SUV_max_ were found between [^68^Ga]Ga-FAPI-04 and [^18^F]F-FDG PET/CT images in both treatment-naïve and post-treatment lesions. However, the average TBR of treatment-naïve lesions was significantly higher on [^68^Ga]Ga-FAPI-04 PET/CT than on [^18^F]F-FDG PET/CT. This result was consistent with the recently study reported by Halil et al. (21). Thus, [^68^Ga]Ga-FAPI-04 PET/CT might be more advantageous than [^18^F]F-FDG PET/CT to improve detecting efficiency in T stage.

The superiority of [^18^F]F-FDG PET/CT is evident in the detection of lymph node and distant metastases in CRC, and the detection of additional metastases can significantly change treatment plans (22, 23). However, several studies demonstrated that the detection of metastatic regional nodes was low/moderate using [^18^F]F-FDG PET/CT, illustrating the limitations of this method (24, 25). Several studies suggested that CRC commonly harbours CAF-expressing FAP. Sugai et al. (26) suggested that high FAP expression is correlated with lymph node metastasis in submucosal invasive CRC. Solano-Iturri et al. (20) observed a significant positive correlation between FAP expression in primary CRC tumours and their corresponding local and distant metastases. Thus, this study examined the reliability of [^68^Ga]Ga-FAPI-04 PET/CT for detecting metastatic CRC lesions. Our results showed that [^68^Ga]Ga-FAPI-04 PET/CT identified additional findings in 41 metastatic and 15 inflammatory lymph nodes of 24 participants with CRC and improved the N staging in these participants. Additionally, [^68^Ga]Ga-FAPI-04 uptake was higher than [^18^F]F-FDG uptake in abdominal and pelvic lymph nodes. However, cervical–supraclavicular and thoracic FDG-avid inflammatory/age-related lymph nodes were FAPI-negative.

According to the M stage, the early detection of isolated metastases in the liver or other sites often improves survival following radical resection (27). Owing to moderate FDG uptake in the liver, [^18^F]F-FDG PET/CT was not the first choice for identifying liver metastasis. Our data revealed that the hepatic background intensity was significantly lower in [^68^Ga]Ga-FAPI-04 PET/CT than that in [^18^F]F-FDG PET/CT, corroborating the findings of previous studies (9, 11, 21). The TBR values of liver metastases were higher in [^68^Ga]Ga-FAPI-04 than those in [^18^F]F-FDG PET/CT in this study. Thus, FAPI-imaging might be advantageous for patients with suspected liver metastases, resulting in a potentially high detection rate. In this study, the smallest lesion detected had a diameter of approximately 0.7 cm. However, Halil et al. (21) found that both the SUV_max_ and TBR values of liver metastases were signifcantly higher in [^18^F]F-FDG than those in [^68^Ga]Ga-FAPI-04 PET/CT. We believe that this issue can be clarified with future studies involving larger and homogeneous cohorts. Reportedly, the peritoneum is another common site of CRC metastasis (28), and the degree of peritoneal metastasis determines the choice of treatment (23, 29). The detection rate of peritoneal metastasis using [^18^F]F-FDG PET/CT is not high, primarily because of intestinal inflammatory uptake, small lesions and other factors, including rare pathological types. [^68^Ga]Ga-FAPI-04 is an active fibroblast-targeted imaging agent, and the development of peritoneal metastases is primarily because of active fibroblasts (28), which is supported by the significantly higher [^68^Ga]Ga-FAPI-04 uptake in peritoneal metastatic lesions compared with [^18^F]F-FDG uptake observed in this study. This result was also consistent with the previous studies (13, 21).

In addition, we found that [^68^Ga]Ga-FAPI-04 uptake was significantly higher than [^18^F]F-FDG uptake in the uterus, which may be attributed to the presence of active fibroblasts in the uterus (30); this suggests that the SUV_max_ of lesions (primary or/and metastatic lesions) located in the pelvic area is affected by high [^68^Ga]Ga-FAPI-04 uptake, a potential limitation of [^68^Ga]Ga-FAPI-04 PET/CT imaging.

This study has several limitations. First, the cohort size was small, and the number of participants with brain and bone metastases was low. Second, the cohort was heterogeneous and included participants with different treatment statuses, which could have affected the SUV_max_ values of lesions. Third, the period of follow-up was set to 3–6 months based on previous experience. Although most of lesions could be observed obvious changes indicating their benign or malignant features, a few lesions might be taken longer to be followed up. Lastly, we could not pathologically confirm all suspicious lesions without considering ethics; thus, neither accurate sensitivity nor specificity could be established. Future studies with larger and homogeneous cohorts are warranted to provide a more comprehensive analysis of the utility of [^68^Ga]Ga-FAPI-04 PET/CT in CRC.

## 5 Conclusion

[^68^Ga]Ga-FAPI-04 PET/CT has several obvious advantages over [^18^F]F-FDG PET/CT, including the detection of lymph nodes and distant metastases, thereby improving the staging of patients with CRC. This improved staging is helpful for the timely revision of clinical treatment strategies and improvement of patients’ prognoses. Additionally, patients might feel more comfortable during [^68^Ga]Ga-FAPI-04 PET/CT as it does not require fasting.

## Data availability statement

The original contributions presented in the study are included in the article/**Supplementary Material**. Further inquiries can be directed to the corresponding authors.

## Ethics statement

The studies involving human participants were reviewed and approved by Institutional Review Board of Beijing Cancer Hospital (no. 2019KT95). The patients/participants provided their written informed consent to participate in this study.

## Author contributions

XL, XW, ZY, and AW contributed to the study conception and design. Synthesis of tracer and image acquisition were performed by SW and YZ. XL, YL, XC, MW, and HZ processed and analysed the data. The first draft of the manuscript was written by XL. XW, ZY, and AW reviewed and revised the manuscript. All authors read and approved the final manuscript.
