# Supplementary material for: Diagnostic value of [68Ga]Ga-FAPI-04 in patients with colorectal cancer in comparison with [18F]F-FDG PET/CT
Source: Front Oncol. 2023 Jan 9;12:1087792. doi: 10.3389/fonc.2022.1087792 (PMC9869033; doi:10.3389/fonc.2022.1087792)
Supplement: Supplementary file 1 [file DataSheet_1.docx]

Supplementary Material

# Supplementary Figures and Tables

## Supplementary Figures


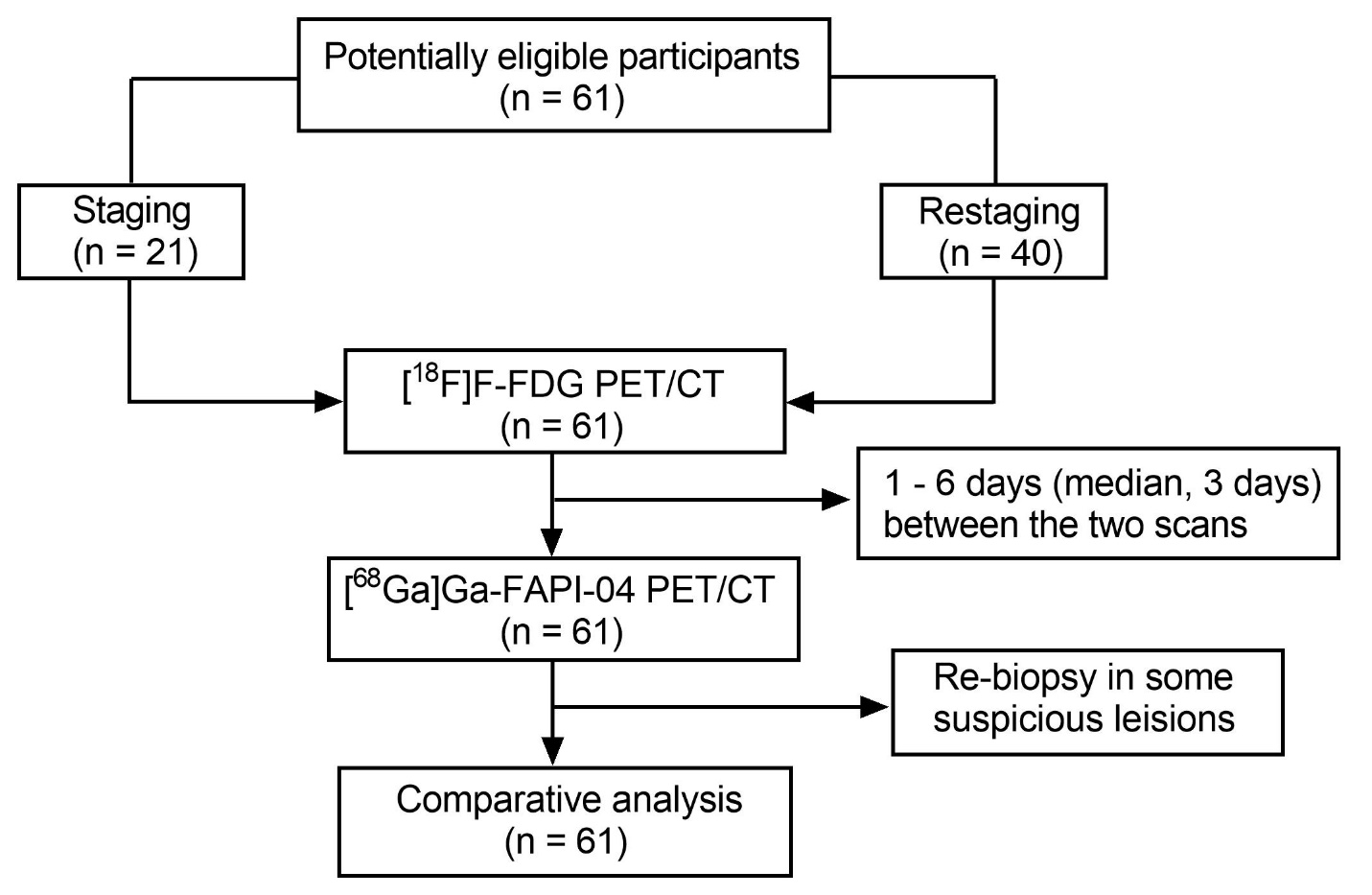


**Supplementary Figure 1.**  Flowchart of participant selection

*[^68^Ga]Ga-FAPI*, gallium 68-fibroblast-activation protein inhibitor; *[^18^F]F-FDG*, fluorine-18 fluorodeoxyglucose; *CT*, computed tomography; *PET*, positron emission tomography

## Supplementary Tables

**Supplementary Table 1.** Comparison of [^18^F]F-FDG PET/CT images of suspicious abdominal lymph nodes reviewed by physicians in group 1

| Reader 2  (M.W.) | Reader 1 (X.C.) | | | Total |
| --- | --- | --- | --- | --- |
|  | − | **+** | −**/+** |  |
| − | 4 | 0 | 1 | 5 |
| **+** | 0 | 17 | 1 | 18 |
| −**/+** | 1 | 0 | 5 | 6 |
| Total | 5 | 17 | 7 | 29 |

−, to be judged negative by visual assessment; +, to be judged positive by visual assessment; −/+, to be judged as ambiguous by visual assessment

The κ value was 0.814 (*P* < 0.001), indicating that the inter-reader agreement was nearly perfect in group 1.

*[^18^F]F-FDG*, fluorine-18 fluorodeoxyglucose; *CT*, computed tomography; *PET*, positron emission tomography

**Supplementary Table 2.** Comparison of [^68^Ga]Ga-FAPI-04 PET/CT images of suspicious abdominal lymph nodes reviewed by physicians in group 2

| Reader 2  (X.W.) | Reader 1 (X.L.) | | | Total |
| --- | --- | --- | --- | --- |
|  | − | **+** | −**/+** |  |
| − | 10 | 0 | 1 | 11 |
| **+** | 0 | 32 | 1 | 33 |
| −**/+** | 0 | 1 | 4 | 5 |
| Total | 10 | 33 | 6 | 49 |

-, to be judged negative by visual assessment; +, to be judged positive by visual assessment; −/+, to be judged as ambiguous by visual assessment

The simple kappa coefficient κ value was 0.875 (*P* < 0.001), indicating that the inter-reader agreement was nearly perfect in group 2.

*[^68^Ga]Ga-FAPI*, gallium 68-fibroblast-activation protein inhibitor; *CT*, computed tomography; *PET*, positron emission tomography

**Supplementary Table 3.** Comparison of [^18^F]F-FDG PET/CT images of suspicious peritoneal lesions reviewed by physicians in group 1

| Reader 2  (M.W.) | Reader 1 (X.C.) | | | Total |
| --- | --- | --- | --- | --- |
|  | − | **+** | −**/+** |  |
| − | 9 | 0 | 1 | 11 |
| **+** | 0 | 15 | 1 | 16 |
| −**/+** | 1 | 0 | 4 | 4 |
| Total | 10 | 15 | 6 | 31 |

−, to be judged negative by visual assessment; +, to be judged positive by visual assessment; −/+, to be judged as ambiguous by visual assessment

The simple kappa coefficient κ value was 0.843 (*P* < 0.001), indicating that the inter-reader agreement was nearly perfect in group 1.

*[^18^F]F-FDG*, fluorine-18 fluorodeoxyglucose; *CT*, computed tomography; *PET*, positron emission tomography

**Supplementary Table 4.** Comparison of [^68^Ga]Ga-FAPI-04 PET/CT images of suspicious peritoneal lesions reviewed by physicians in group 2

| Reader 2  (X.W.) | Reader 1 (X.L.) | | | Total |
| --- | --- | --- | --- | --- |
|  | − | **+** | −**/+** |  |
| − | 13 | 0 | 1 | 14 |
| **+** | 0 | 22 | 2 | 24 |
| −**/+** | 0 | 0 | 4 | 4 |
| Total | 13 | 22 | 7 | 42 |

−, to be judged negative by visual assessment; +, to be judged positive by visual assessment; −/+, to be judged as ambiguous by visual assessment

The simple kappa coefficient κ value was 0.892 (*P* < 0.001), indicating that the inter-reader agreement was nearly perfect in group 2.

*[^68^Ga]Ga-FAPI*, gallium 68-fibroblast-activation protein inhibitor; *CT*, computed tomography; *PET*, positron emission tomography

**In conclusion, the inter-reader agreement in groups 1 and 2 was nearly perfect.**
